# Attention Deficit Hyperactivity Disorder as a determinant of motorcycle accidents in Fars province of Iran 

**Published:** 2019-08

**Authors:** Arash Mani, Seyed Taghi Heydari, Yaser Sarikhani, Mehrdad Vossoughi, Kamran Bagheri Lankarani

**Affiliations:** ^*a*^Research Center for Psychiatry and Behavior Sciences and Community Based Psychiatric Care Research Center, Shiraz University of Medical Sciences, Shiraz, Iran.; ^*b*^Health Policy Research Center, School of Medicine, Shiraz University of Medical Sciences, Shiraz, Iran.; ^*c*^Student Research Committee, Shiraz University of Medical Sciences, Shiraz, Iran.; ^*d*^Department of Dental Public Health, Oral and Dental Disease Research Center, School of Dentistry, Shiraz University of Medical Sciences, Shiraz, Iran.; ^*e*^Health Policy Research Center, School of Medicine, Shiraz University of Medical Sciences, Shiraz, Iran.

## Abstract

**Background:**

Road traffic accident is a serious public health problem in the world. Iran is among the countries with the highest rate of traffic accident causing high mortality and morbidity. Although the number of motorcycle drivers is less than car drivers in Iran, a major part of mortality and morbidity belongs to motorcycle drivers especially in rural areas. Attention Deficit Hyperactivity Disorder is one of the predicting factors for risky behaviors and accidents among the motorcyclists. The aim of this study was to investigate the relationship between motorcycle accidents and attention deficit hyperactivity disorder in Shiraz, Iran.

**Methods:**

In this cross sectional study was done in 2017 total number of 1195 motorcyclist from Fars province were included. In this study we collected the data using the Persian version of Conners Adult Attention Deficit Hyperactivity Disorder (ADHD) Rating Scales (CAARS) 30-item questionnaire and a checklist designed by the researchers for demographic variables. SPSS 22 was used to analyze the data.

**Results:**

All the study participants were male and the mean age was 28.28±8.56 years. 16.7% of the motorcyclists had motorcycle driving license, 25.4% and 23.3% had the experience of driving fines and history of accident respectively. The mean score of hyperactivity disorder was 32.3±16.8. The mean score of ADHD was higher among the motorcyclists with the history of accident in the past year (P=0.018), those with speeding over the limit, those with maneuvering while driving, drivers who used mobile phone during driving, and those who had more pleasure while driving motorcycle (p<0.001).

**Conclusions:**

Findings of this study indicated that traffic accidents increased with the higher scores of ADHD in motorcyclists, and hyperactivity may be considered as a risk factor of driving risky behaviors such as exceeding the speed limits, illegal overtaking, and maneuvering while driving and finally accidents of motorcyclists.

**Keywords:**

Traffic Accident, Motorcyclists, Attention Deficit/Hyperactivity Disorder

